# Supplementary material for: Structured Prompt Interrogation and Recursive Extraction of Semantics (SPIRES): a method for populating knowledge bases using zero-shot learning
Source: Bioinformatics. 2024 Feb 21;40(3):btae104. doi: 10.1093/bioinformatics/btae104 (PMC10924283; doi:10.1093/bioinformatics/btae104)
Supplement: btae104_Supplementary_Data [file btae104_supplementary_data.pdf]

Supplementary Data

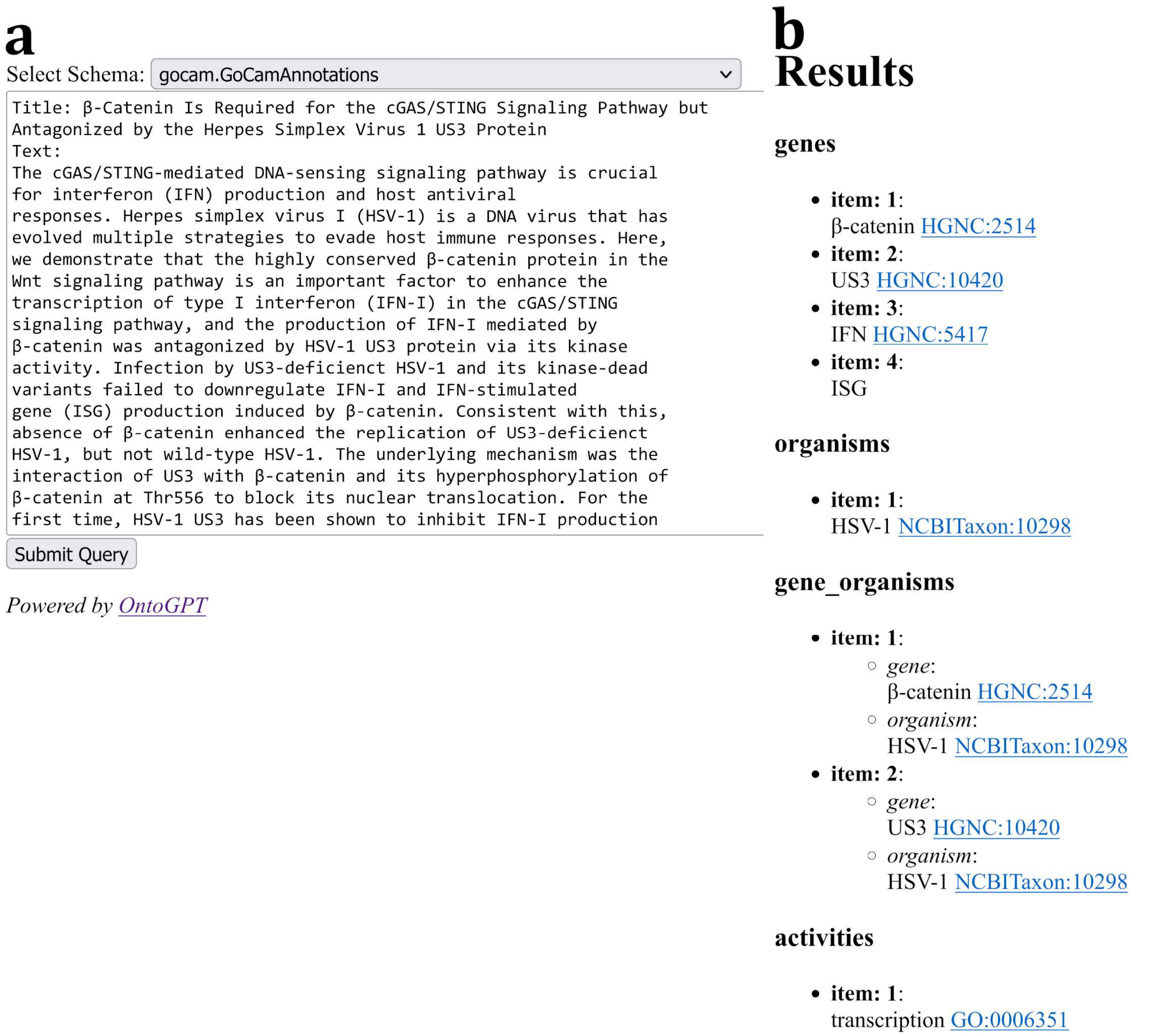

Fig. S1. Screenshot of web-ontogpt. (a) Form entry page, allowing selection of schema, plus input text. (b) Sample of results as structured object rendered as nested HTML. Note that both input text and results are truncated for brevity.

Table S1. Resources used for grounding during evaluation of SPIRES with relations in the BC5CDR test corpus. These resources were used for initial annotation and are subsequently normalized to MeSH. Annotations from the Gilda text entity normalization tool are retrieved through its API (`http://grounding.indra.bio/apidocs`) using the Ontology Access Kit.

Entity type	Resource	Prefix	Source
Chemical	Medical Subject Headings 2022	MESH	[Lipscomb, 2000]
	Chemical Entities of Biological Interest	CHEBI	[Hastings et al., 2016]
	National Cancer Institute Thesaurus	NCIT	[Sioutos et al., 2007]
	Mapping of Drug Names and MeSH 2022	MDM	[Lipscomb, 2000]
	DrugBank	DRUGBANK	[Wishart et al., 2018]
	Gilda	N/A	[Gyori et al., 2022]
Disease	Medical Subject Headings 2022	MESH	[Lipscomb, 2000]
	Mondo Disease Ontology	MONDO	[Mungall et al., 2017]
	Human Phenotype Ontology	HP	[Köhler et al., 2021]
	National Cancer Institute Thesaurus	NCIT	[Sioutos et al., 2007]
	Human Disease Ontology	DOID	[Schriml et al., 2019]
	Medical Dictionary for Regulatory Activities	MEDDRA	[Brown et al., 1999]

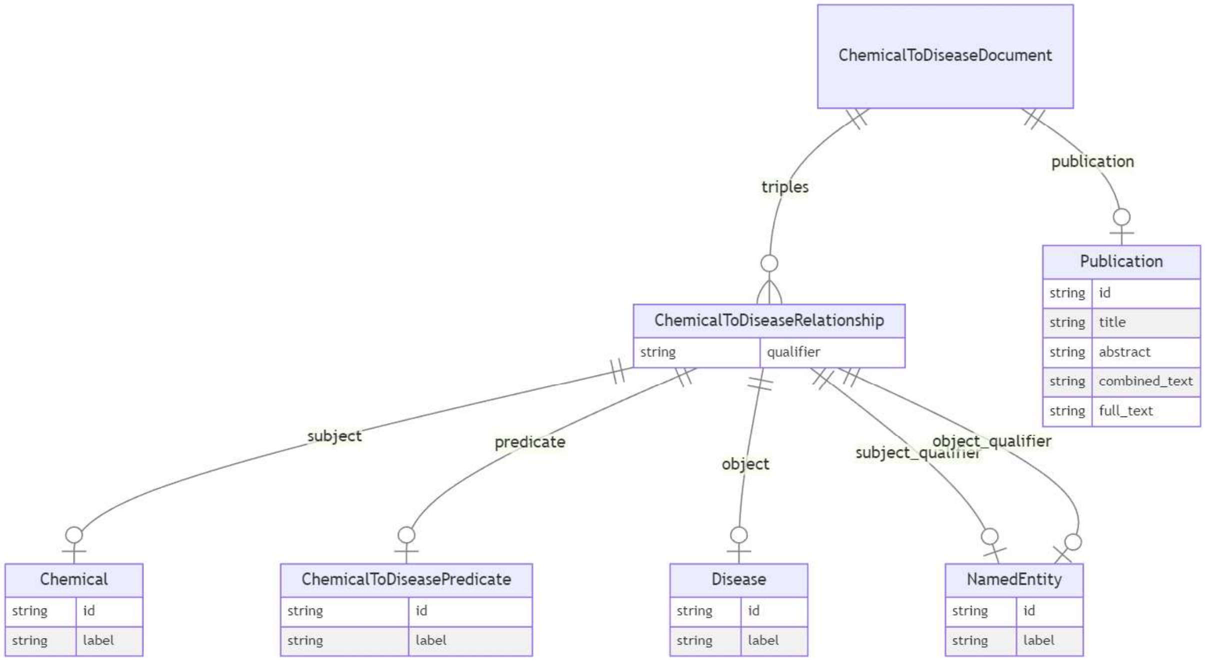

Fig. S2. Chemical to Disease (CTD) schema (available from `https://w3id.org/ontogpt/ctd`).

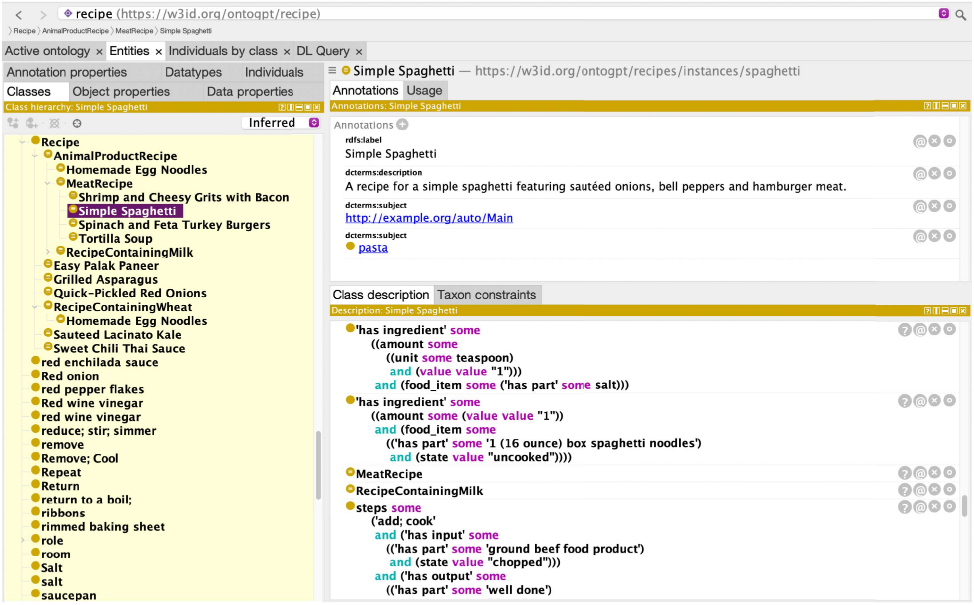

Fig. S3. Screenshot of extracted recipes in a merged OWL file from the Protege ontology editor [Musen and Protégé Team, 2015]. The "Simple Spaghetti" recipe is correctly classified under MeatRecipe, due to the presence of an ingredient that is classified as a meat-based product in FOODON. The right hand panel shows OWL logical axioms for the recipe, including its ingredients, and the steps involved.

Table S2. MeSH identifiers used to define value sets during evaluation of SPIRES with relations in the BC5CDR test corpus. All identifiers in this table were treated as root nodes of a hierarchy, i.e., the value sets include all child MeSH terms.

Entity type	MeSH identifier	MeSH term
Chemical	D602	Amino Acids, Peptides, and Proteins
	D1685	Biological Factors
	D2241	Carbohydrates
	D4364	Pharmaceutical Preparations
	D6571	Heterocyclic Compounds
	D7287	Inorganic Chemicals
	D8055	Lipids
	D9706	Nucleic Acids, Nucleotides, and Nucleosides
	D9930	Organic Chemicals
	D11083	Polycyclic Compounds
	D13812	Therapeutics
	D19602	Food and Beverages
	D45424	Complex Mixtures
	D45762	Enzymes and Coenzymes
	D46911	Macromolecular Substances
Disease	D001423	Bacterial Infections and Mycoses
	D001523	Mental Disorders
	D002318	Cardiovascular Diseases
	D002943	Circulatory and Respiratory Physiological Phenomena
	D004066	Digestive System Diseases
	D004700	Endocrine System Diseases
	D005128	Eye Diseases
	D005261	Female Urogenital Diseases and Pregnancy Complications
	D006425	Hemic and Lymphatic Diseases
	D007154	Immune System Diseases
	D007280	Disorders of Environmental Origin
	D009057	Stomatognathic Diseases
	D009140	Musculoskeletal Diseases
	D009358	Congenital, Hereditary, and Neonatal Diseases and Abnormalities
	D009369	Neoplasms
	D009422	Nervous System Diseases
	D009750	Nutritional and Metabolic Diseases
	D009784	Occupational Diseases
	D010038	Otorhinolaryngologic Diseases
	D010272	Parasitic Diseases
	D012140	Respiratory Tract Diseases
	D013568	Pathological Conditions, Signs and Symptoms
	D014777	Virus Diseases
	D014947	Wounds and Injuries
	D017437	Skin and Connective Tissue Diseases
	D052801	Male Urogenital Diseases
	D064419	Chemically-Induced Disorders

Table S3. Results for named entity recognition evaluation of SPIRES on chemical and disease entities in the BC5CDR corpus. The chunking strategy was not used in this evaluation. Grounding was performed against MeSH only - further accuracy may be afforded by use of alternate ontology annotators such as CHEBI or MONDO for chemical and disease, respectively.

Entity type	Model	F-score	Precision	Recall
Chemical	GPT-3.5-turbo	69.70	0.89	0.57
	GPT-4	73.69	0.85	0.65
Disease	GPT-3.5-turbo	61.70	0.87	0.48
	GPT-4	69.70	0.88	0.56
